# Supplementary material for: Cloud BioLinux: pre-configured and on-demand bioinformatics computing for the genomics community
Source: BMC Bioinformatics. 2012 Mar 19;13:42. doi: 10.1186/1471-2105-13-42 (PMC3372431; doi:10.1186/1471-2105-13-42)
Supplement: Additional file 1 — Supplementary 1 Cloud BioLinux software documentation in the form of a mini, self-contained website. Users need to download and uncompress the .zip file, and open through a web browser the "index.html" file available on the main directory. (ZIP 1823 kb). [file 1471-2105-13-42-S1.ZIP › Cloud-BioLinux-Package-Documentation/docs/hmmindex.html]

Bio-Linux Software Documentation Pages

Back to search form

## hmmindex

|  |  |
| --- | --- |
| Name | hmmindex |
| Description | **hmmindex** is part of the HMMer package. HMMER is an implementation of profile hidden Markov models (profile HMMs) for biological sequence analysis. Profile HMMs are statistical models of multiple sequence alignments. They capture position-specific information about how conserved each column of the alignment is, and which residues are likely.  **hmmindex** - create a binary GSI index for an HMM database  Sample data on Bio-Liniux for the HMMER package can be found in  `/usr/software/sampledata/hmmer_demo_data`  **References:**  Krogh, A., Brown, M., Mian, I. S., Sjolander, K., and Haussler, D. (1994). Hidden Markov models in computational biology: Applications to protein modeling. J. Mol. Biol., 235:1501 1531.[Entrez]    Durbin, R., Eddy, S. R., Krogh, A., and Mitchison, G. J. (1998). Biological Sequence Analysis: Probabilistic Models of Proteins and Nucleic Acids. Cambridge University Press, Cambridge UK.    Eddy, S. R. (1998). Profile hidden Markov models. Bioinformatics, 14:755 763.[Entrez] |
| Homepage | http://hmmer.wustl.edu |
| Remote Documentation | ftp://ftp.genetics.wustl.edu/pub/eddy/hmmer/CURRENT/Userguide.pdf |
